# FGF23 and its role in X-linked hypophosphatemia-related morbidity

**DOI:** 10.1186/s13023-019-1014-8

**Published:** 2019-02-26

**Authors:** Signe Sparre Beck-Nielsen, Zulf Mughal, Dieter Haffner, Ola Nilsson, Elena Levtchenko, Gema Ariceta, Carmen de Lucas Collantes, Dirk Schnabel, Ravi Jandhyala, Outi Mäkitie

**Affiliations:** 1 Centre for Rare Diseases, Aarhus University Hospital, Aarhus, Denmark; 2 Royal Manchester Children’s Hospital, Manchester, UK; 3 Hannover Medical School, Hannover, Germany; 4 Karolinska Institutet, Stockholm, Sweden and Örebro University, Örebro, Sweden; 5 Katholieke Universiteit Leuven, Leuven, Belgium; 6 Hospital Universitario Materno-Infantil Vall d’Hebron, Universitat Autonoma de Barcelona, Barcelona, Spain; 7 Hospital Niño Jesús, Madrid, Spain; 8 University Children’s Hospital of Berlin, Berlin, Germany; 9 Medialis Ltd, Banbury, UK; 10 Children’s Hospital, University of Helsinki and Helsinki University Hospital, Helsinki, Finland

**Keywords:** X-linked hypophosphatemia (XLH), fibroblast growth factor 23 (FGF23), phosphate regulating endopeptidase homolog, X-linked (PHEX), hypophosphatemia, vitamin D deficiency, rickets, osteomalacia, bone dysplasia, ectopic calcification, muscle weakness, dental abscess, hearing impairment

## Abstract

**Background:**

X-linked hypophosphatemia (XLH) is an inherited disease of phosphate metabolism in which inactivating mutations of the *Phosphate Regulating Endopeptidase Homolog, X-Linked* (*PHEX*) gene lead to local and systemic effects including impaired growth, rickets, osteomalacia, bone abnormalities, bone pain, spontaneous dental abscesses, hearing difficulties, enthesopathy, osteoarthritis, and muscular dysfunction. Patients with XLH present with elevated levels of fibroblast growth factor 23 (FGF23), which is thought to mediate many of the aforementioned manifestations of the disease. Elevated FGF23 has also been observed in many other diseases of hypophosphatemia, and a range of animal models have been developed to study these diseases, yet the role of FGF23 in the pathophysiology of XLH is incompletely understood.

**Methods:**

The role of FGF23 in the pathophysiology of XLH is here reviewed by describing what is known about phenotypes associated with various PHEX mutations, animal models of XLH, and non-nutritional diseases of hypophosphatemia, and by presenting molecular pathways that have been proposed to contribute to manifestations of XLH.

**Results:**

The pathophysiology of XLH is complex, involving a range of molecular pathways that variously contribute to different manifestations of the disease. Hypophosphatemia due to elevated FGF23 is the most obvious contributor, however localised fluctuations in tissue non-specific alkaline phosphatase (TNAP), pyrophosphate, calcitriol and direct effects of FGF23 have been observed to be associated with certain manifestations.

**Conclusions:**

By describing what is known about these pathways, this review highlights key areas for future research that would contribute to the understanding and clinical treatment of non-nutritional diseases of hypophosphatemia, particularly XLH.

## Background and Introduction

**Fig. 1:**
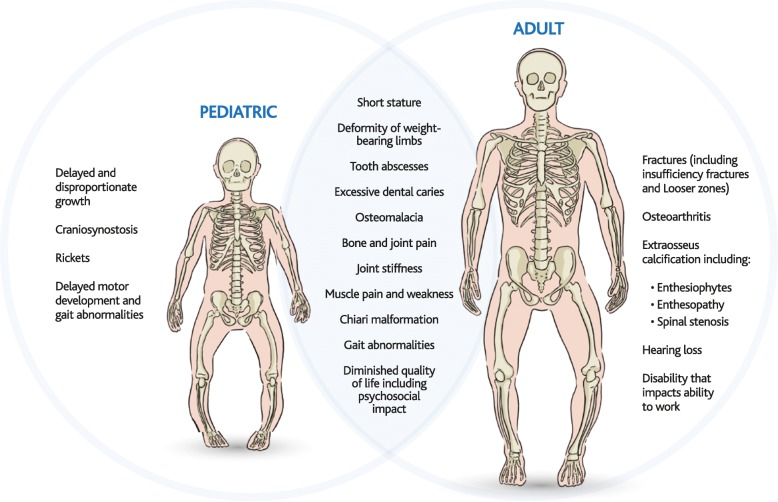
Symptomatology and pathophysiology of XLH. The signs, symptoms, sequelae, and long-term consequences of XLH in paediatric (left) and adult (right) patients

X-linked hypophosphatemia (also known as X-linked hypophosphatemic rickets, XLH; OMIM: #307800) is an inherited disease of phosphate metabolism, where inactivating mutations of the *Phosphate Regulating Endopeptidase Homolog, X-Linked* (*PHEX*, OMIM: #300550) gene lead to local and systemic effects. XLH affects approximately 1:20,000 individuals [1] who experience a diverse range of medical problems that are depicted in Fig. 1, and include impaired growth, rickets, osteomalacia, bone abnormalities, bone pain, spontaneous dental abscesses, hearing difficulties, enthesopathy, osteoarthritis, and muscular dysfunction [2, 3].

*PHEX* is predominantly expressed in osteoblasts and codes for an enzyme that degrades local small integrin-binding ligand, N-linked glycoproteins (SIBLING proteins), particularly osteopontin (OPN) [4], and suppresses serum levels of the phosphatonin, fibroblast growth factor 23 (FGF23). Despite being an enzyme, PHEX is thought to affect expression [5] rather than degradation of FGF23 [6, 7].

Downregulation of PHEX in XLH increases skeletal OPN deposition which contributes to local inhibition of mineralisation [4]. Meanwhile, elevated levels of serum FGF23 increase urinary phosphate excretion by downregulating renal sodium-phosphate transporters, and limit intestinal phosphate absorption by restricting active vitamin D synthesis to levels that are abnormally low or normal despite hypophosphatemia [8] .

Since phosphate insufficiency and inappropriately low levels of calcitriol [also known as 1,25(OH)_2_D or active vitamin D] contribute to many symptoms of XLH, conventional therapy involves supplementation with oral phosphate and calcitriol or calcitriol analogues (commonly alfacalcidol). This can correct lower limb deformities, promote growth, and improve oral health [9], with earlier treatment leading to better results [10]. However, conventional therapy insufficiently corrects the biochemistry and symptoms of XLH, and can further increase serum FGF23 levels [8, 11–13]. Conventional therapy has also been associated with adverse effects including secondary hyperparathyroidism, nephrocalcinosis, nephrolithiasis, and cardiovascular abnormalities [14].

Although hypophosphatemia is the primary link between elevated FGF23 and the pathophysiology of XLH, FGF23 has recently been proposed to also contribute to XLH *via* other molecular mechanisms [7, 15].

**Fig. 2:**
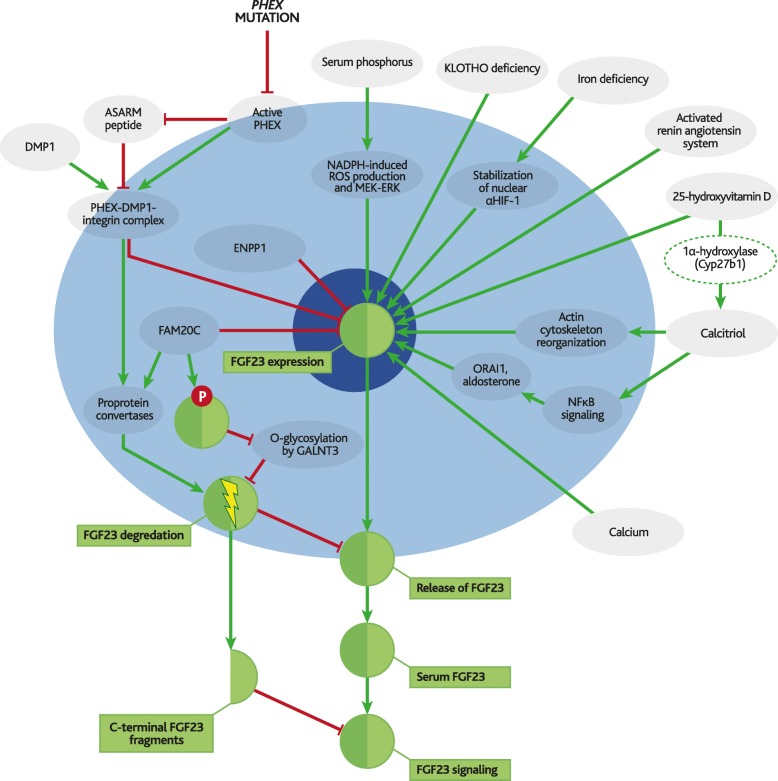
Regulation of FGF23 expression and secretion in XLH. Inactivating mutations in *PHEX* increase fibroblast growth factor 23 (FGF23) expression by increasing levels of acidic serine aspartate-rich-MEPE-associated protein (ASARM) peptide. This leads to increased release of FGF23 into the serum, and increased levels of FGF23-mediated signalling. These processes are also regulated by a wide range of other mechanisms. Green lines indicate upregulation and red lines indicate repression. For simplification, feedback loops have been represented as linear pathways centred around FGF23

This review describes the central role of FGF23 in XLH pathophysiology, outlining evidence that links upregulation of FGF23 to manifestations of XLH through various molecular pathways (outlined in Fig. 2). FGF23 is introduced along with its direct regulators and receptors, followed by a brief discussion of the dysregulation of serum FGF23 in various diseases of hypophosphatemia; animal models of these diseases are also described since they are essential for understanding molecular mechanisms involved in the pathology of XLH. Finally, the manifestations of XLH are grouped by molecular mechanism and discussed, with any potential involvement of FGF23 highlighted.

### Regulation of serum FGF23

The *FGF23* gene is located on chromosome 12 and codes for a 251-amino acid, 32 kDa pro-protein. Although FGF23 is predominantly expressed in and secreted by osteocytes and osteoblasts, lower levels of FGF23 expression have been detected in rodents in many non-bone tissues, including teeth and brain [16–18].

A 24-amino acid signalling peptide is cleaved from FGF23 post-translationally and directs active FGF23 protein (227 amino acids) to the Golgi apparatus for secretion. Some active FGF23 is further cleaved during secretion, and the resulting C- and N-terminal fragments are then released from the cell along with the remaining active FGF23; these FGF23 fragments are not thought to have any innate biological activity [19, 20] . FGF23 can either act locally or enter the bloodstream to interact with distant cell surface receptors. The molecular pathways involved in regulation of these processes are complex, and therefore are only briefly depicted in Fig. 2 and summarised below.

### Factors that regulate FGF23 expression

Expression of FGF23 is predominantly regulated by serum phosphate and calcitriol [21]. Phosphate-induced elevation of serum FGF23 mostly occurs in bone [22]. The nature of this “phosphate sensing” mechanism is yet to be fully elucidated, but has been proposed to involve nicotinamide adenine dinucleotide phosphate (NADPH)-induced production of reactive oxygen species (ROS), and the mitogen-activated protein kinase kinase-extracellular signal-regulated kinases (MEK-ERK) pathway [23, 24]. Other molecular mechanisms that have been associated with FGF23 expression include FAM20C [25], ENPP1 [26] and DMP1 [27], as well as the presence of SIBLING protein-derived acidic serine aspartate-rich-MEPE-associated protein (ASARM) peptides [28].

Recent additions to the long list of factors proposed to affect FGF23 expression include actin cytoskeleton reorganisation, NFκB signalling [29], aldosterone [30], *ORAI1* [31], changes in calcium concentrations, activated renin angiotensin system, KLOTHO [32], and local osteoblastic conversion of 25(OH)D to calcitriol [33].

### Factors that regulate FGF23 cleavage

Degradation of FGF23 has been proposed to be mediated by furin [19] and/or proprotein convertase, subtilisin/kexin-type 5/6 (PC5/6) [34], and to be inhibited by O-glycosylation at the site of proteolysis by polypeptide N-acteylgalatosaminyltransferase 3 (GalNAcT3), which is encoded by the *GALNT3* gene [35, 36]. Homozygous inactivating mutations in *GALNT3* result in low levels of intact FGF23 and familial tumoral calcinosis syndrome, a condition characterised by hyperphosphatemia and extraskeletal calcifications [37]. There is also evidence that O-glycosylation can be blocked by FAM20C-mediated phosphorylation [19], and that FGF23 can be cleaved by proprotein convertases [38], although these findings have been challenged [34].

A recent study found that both expression and cleavage of FGF23 was promoted by iron deficiency and inflammation, so that secretion of C-terminal fragments was upregulated without significantly affecting serum concentrations of active FGF23 [39].

While it is important to appreciate the complexity of FGF23 regulation and to recognise that *PHEX* mutations disrupt a finely balanced system, many of the aforementioned pathways have already been well-reviewed [40, 41] and a more detailed description of them is beyond the scope of the current article.

### FGF23 receptors and signalling

**Fig. 3:**
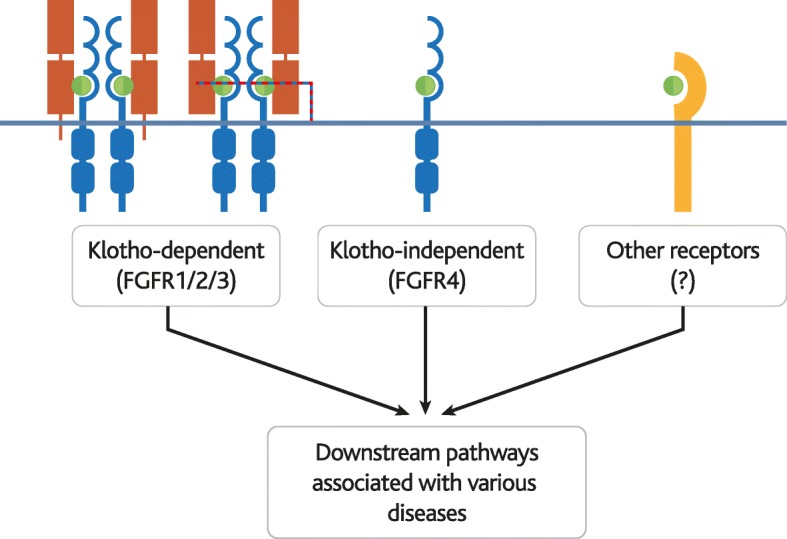
FGF23-receptor interactions. Schematic diagram of known and potential interactions between FGF23 (green circle) and its various receptors (blue and yellow). (left) FGF23 is known to bind in a KLOTHO (red)-dependent fashion to FGFRs 1, 2, and 3, (centre) and to bind to FGFR4 independently of KLOTHO. (right) KLOTHO-independent interactions with other receptors have also been proposed

The poor *in vitro* affinity of FGF23 for its receptors made it seem like an unlikely candidate for the then putative phosphatonin [42]. However, poor receptor-ligand affinity is overcome *in vivo* via utilisation of co-receptors, particularly α-KLOTHO (KLOTHO), which is schematically represented in Fig.3. Receptors for FGF23 include FGF receptor (FGFR)1, FGFR2, FGFR3, and FGFR4, and the expression of these receptors varies between cell types [43, 44]. In addition, FGF23 has unusually poor affinity for heparan sulfate (HS) that allows it to diffuse through the HS-rich extracellular matrix more readily than other FGFs, and to signal in an endocrine fashion [45].

KLOTHO greatly enhances the affinity of FGF23 for FGFR1 and FGFR3 [43] and is required for many functions of FGF23. The close relationship between KLOTHO and FGF23 is highlighted in KLOTHO-deficient (*Klotho*^-/-^) mice, which display a very similar phenotype to *Fgf23*^-/-^ mice, even when they are forced to overexpress FGF23 [43, 46, 47]. In addition, it was demonstrated that the shed extracellular domain of α-KLOTHO (α-KlOTHO^ecto^) serves as a non-enzymatic molecular scaffold for FGF23 hormone signalling. α-KlOTHO^ecto^ is able to form a 1:1:1 ternary complex together with FGF23 and FGFR1 by implementing FGF23-FGFR1 proximity and conferring stability. As demonstrated for membrane bound α-KLOTHO, this is followed by heparan sulfate facilitated dimerisation of two ternary complexes promoting FGF23 signalling (Fig. 3).

KLOTHO expression was originally thought to limit FGF23 activity and to be restricted to the kidney, parathyroid gland and choroid plexus [48]. However, recent reports of widespread KLOTHO expression [48], α-KlOTHO^ecto^ mediated FGFR activation, and KLOTHO-independent functions of FGF23 [49] have expanded the proposed scope of FGF23 activity.

Post-cleavage C-terminal fragments of FGF23 have also been shown to block formation of FGF23-FGFR-KLOTHO complexes and to improve hypophosphatemia, which adds another regulatory level to FGF23 signalling [50].

### Serum FGF23 and non-nutritional diseases of hypophosphatemia

Serum FGF23 is elevated in many non-nutritional diseases of hypophosphatemia, and the main characteristics that lead to differential diagnosis of these diseases are outlined in Table 1.

**Table 1:** Non-nutritional diseases of hypophosphatemia

Disease of interest	Abbreviation	MIM#	Gene affected	FGF23 levels	Hypophosphatemia	Calcitriol levels	Osteomalacia/ rickets/stunted growth	Craniofacial abnormalities	Dental abscesses	Enthesopathies	Nephrocalcinosis	Hearing loss	Hypertension	References
X-linked hypophosphatemia	XLH	307800	*PHEX*	↑	Y	Normal or low	1^st^	1^st^	1^st^	1^st^	2^nd^	1^st^	Y	[3, 9]
Autosomal dominant hypophosphatemic rickets	ADHR	193100	*FGF23*	↑	Y	Normal or low	1^st^	N/A	1^st^	N/A	N/A	N/A	N/A	[51]
Autosomal recessive hypophosphatemic rickets 1	ARHR1	241520	*DMP1*	↑	Y	N/A	1^st^	1^st^	1^st^	Y	N/A	N/A	Y	[52]
Autosomal recessive hypophosphatemic rickets 2	ARHR2	613312	*ENPP1*	↑	Y	N/A	1^st^	N/A	1^st^	N/A	N/A	Has been associated with GACI	1^st^ [GACI, [248]]	[26]
Autosomal recessive hypophosphatemic rickets 3 (Raine Syndrome)	ARHR3	259775	*FAM20C*	↑	Y	N/A	Not mentioned in the very few cases that survived to preadolescence	1^st^	N/A	N/A	N/A	N/A	N/A	[25]
Tumor-induced osteomalacia	TIO	N/A	N/A	↑	Y	Low	1^st^	No	N/A	N/A	N/A	N/A	N/A	[249, 250]
Hypophosphatemic rickets with hypercalciuria	HHRH	241530	*SLC34A3* (NPT2C)	-	Y	Elevated	1^st^	N/A	N/A	N/A	1^st^ (hypercalciuria)	N/A	N/A	[251]

↑: elevated, -: normal, N: no, Y: yes, 1^st^: primary effect, 2^nd^: secondary effect (caused by conventional treatment), N/A = not assessed, MIM = Mendelian Inheritance in Man

FGF23 was originally identified for its role in phosphate metabolism when mutated *FGF23* was found in patients with autosomal dominant hypophosphatemic rickets (ADHR) [51], and FGF23 was identified as the causal agent in tumour-induced osteomalacia (TIO). Indeed, most inherited forms of hypophosphatemia are caused by mutations that directly increase serum concentrations of FGF23 and/or the activity of its receptors.

The three forms of autosomal recessive hypophosphatemic rickets (ARHR) result from mutations in *DMP1* [ARHR1, [52]], *ENPP1* [ARHR2, [26]], and *FAM20C* [ARHR3, [25]], while hypophosphatemic rickets and hyperparathyroidism (HRHPT) is caused by mutations that upregulate KLOTHO expression [53].

A key exception to the rule is hereditary hypophosphatemic rickets with hypercalciuria (HHRH) in which patients show suppressed or low-normal FGF23 levels. HHRH is caused by mutations in the renal phosphate transporter *NPT2C* [also known as *NaPi-IIc* or *solute carrier family 34 member 3 (SLC34A3)*]. Phosphate-independent effects of FGF23 can therefore be identified by comparing the pathophysiology of patients with HHRH to those with FGF23-high hypophosphatemias [54] (Table 1).

TIO is an interesting example of an FGF23-mediated hypophosphatemia, as this acquired disease is caused by FGF23-secreting tumours, whose total resection is completely curative [55]. Symptoms of TIO can therefore be unambiguously attributed to over-expression of FGF23 and/or other tumour-secreted phosphatonins, without potential confounding contributions to the clinical phenotype from mutated genes.

Finally, XLH is the most common form of non-nutritional hypophosphatemia. The mechanisms by which *PHEX* mutations lead to elevated FGF23 levels remain poorly understood and have been attributed to increased expression [5] and reduced degradation [38, 40] of FGF23.

### *PHEX* mutations and FGF23 regulation in XLH

**Fig. 4:**
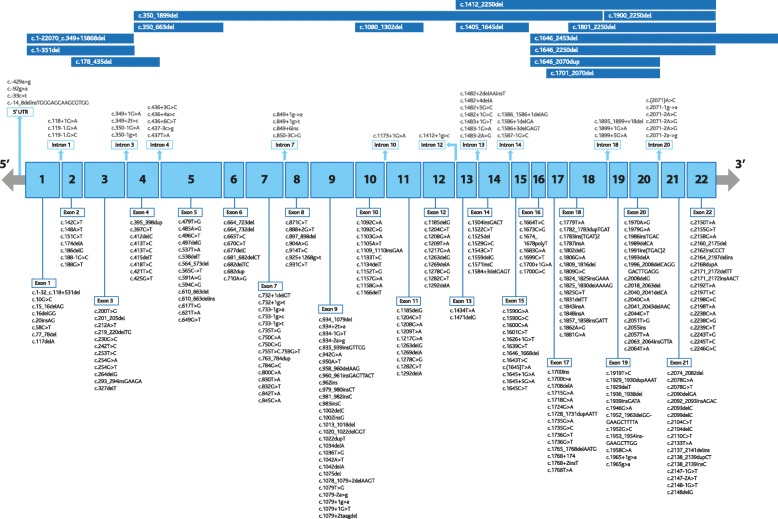
Mutation analysis of human *PHEX*. Mutations that span multiple exons (top section) are represented by lines, while intron-specific (middle section) and exon-specific (lower section) mutations are clustered by loci. Affected nucleotides are numbered. A, adenine; C, cytosine; G, guanine; T, thymine; del, deletion mutations; dup, duplication mutations; ins, insertion mutations; delins, combination deletion/insertion mutations; >, substitution mutations [56]

Around 350 different *PHEX* mutations have been identified to date, including nonsense, missense, frameshift, splice site, deletion, and duplication mutations [56] – the mutations are represented in Fig. 4. Mutations have been observed to affect each of the 22 *PHEX* exons, as well as intronic splice sites [57–59] and the 5’ untranslated region [57].

Identifying consistent genotype-phenotype relationships in patients with mutations affecting similar genetic loci would indicate connections between those *PHEX* loci and specific phenotypes. The presence or absence of a correlation between such mutations and serum levels of FGF23 could further elucidate the role of *PHEX* in regulating FGF23, and/or the roles of *PHEX* and FGF23 in the pathophysiology of XLH. Indeed, specific mutations have been associated with specific manifestations of XLH [56], and attempts have been made to connect serum levels of FGF23 to severity of XLH [8].

*PHEX* mutations can lead to retention of PHEX protein in the endoplasmic reticulum [60] and/or expression of truncated PHEX proteins which may retain some functions of the full-length protein [61, 62]. Identifying direct functions of PHEX that may be retained in truncated proteins would therefore contribute to our understanding of the aetiology of XLH.

One mechanism by which the full-length PHEX protein has been proposed to regulate serum FGF23 is indirect cleavage by proprotein convertases [6, 40]. The proprotein convertase, subtilisin/kexin-type 2 (PC2) has been reported to be upregulated by PHEX, to cleave FGF23 directly [40], and to promote formation of PHEX-DMP1-integrin complexes that suppress FGF23 when activated by neuroendocrine protein 7B2 (7B2•PC2) [38]. However, the potential for a direct interaction between 7B2•PC2 and FGF23 may be confounded by an apparent lack of potential for physical contact between the two proteins [34]. Interactions between 7B2•PC2 and the FGF23-regulator FAM20C may [63] or may not [34] also affect interpretation of these studies.

However, studies searching for genotype-phenotype correlations in XLH patients have so far failed to identify significant correlations [64–67]. Reaching statistical significance in these studies is complicated by the rarity of the disease, the sheer diversity of mutations in XLH patients and the impact of conventional treatment on the natural course of the disease [65].

Although mutations can be grouped by locus [64] or by mutation type [66], larger data sets are ultimately required to establish specific genotype-phenotype correlations. The observation that disease severity may vary considerably in affected members of the same family indicates that there are other modifying factors and that a clear genotype-phenotype correlation may be difficult to identify [68].

### Animal models of XLH

Despite the lack of obvious genotype-phenotype correlation in individuals with *PHEX* mutations, phenotypic differences have been observed between various animal models of XLH, which are easier to study but are not precisely representative of the patient population.

Mouse models possessing at least six different mutations of the *Phex* gene (Gy, Hyp, Hyp-Duk, Hyp-2J, Ska1 and Jrt) have been used to study XLH and are described in Table 2. The phenotypes of these mice vary depending on the specific *Phex* mutation and the mouse strain. Although these differences are rarely discussed in published works, they can shed light on FGF23-independent roles of PHEX in XLH pathology and resolve apparent contradictions in the literature.

**Table 2:** Genotypic and phenotypic summaries of XLH animal models

Name	Mutation size and type	Exon(s) affected	Phex-specific	Serum FGF23	Serum phosphate	Osteomalacia/ rickets/short stature	Craniofacial abnormalities	Hearing loss	References
Gy	Deletion (160-190 kb)	UTR+1-3	N	↑	↓	Y	N	Y	[73]
Hyp	Deletion (58 kb)	15-22+UTR	N	↑	↓	Y	Y	N	[71]
Hyp-Duk	Deletion (30 kb)	13-14	Y	↑	↓	Y	Y	Y	[62]
Hyp-2J	Deletion (7.3 kb)	15	Y	↑	↓	Y	Y	N	[62]
Ska1	Point mutation (1 bp)	8	Y	↑	↓	Y	?	?	[75]
Jrt	Point mutation (1 bp)	14	Y	↑	↓	Y	?	?	[76]

The size and type of mutations, the exons affected and whether or not the mutations are restricted to the *Phex* coding region are presented alongside the phenotypic profile of the mice. Y, yes/present; N, no/absent; ?, not reported; UTR, untranslated region

### *Phex*-non-specific animal models of XLH

The first two mouse models of XLH were named Hyp and Gy [69, 70]. Although both mice displayed hypophosphatemia and broadly similar phenotypes, Gy mice additionally displayed inner ear abnormalities and male sterility. The two models were originally thought to possess mutations in related X-linked genes, but Gy and Hyp have since been identified as mutations that both ablate the *Phex* gene (Table 2). While Hyp mice have mutations that affect exon 15 and 10 kb of downstream intergenic sequences, Gy mice contain large deletions of *Phex* exons 1-3 [71, 72]. The Gy deletion also extends upstream into the neighbouring spermine synthase (SmS) gene, which has been associated with hearing loss and infertility, thus confounding Gy mice as a model of XLH-related hearing loss [73].

Forced expression of human transgenic FGF23 has been shown to rescue the bone phenotype of Hyp mice, but not their hypophosphatemia [74]. This result may be due to phosphate-independent effects of FGF23 on bone, or the Hyp mutation affecting expression of other genes or signal peptides involved in phosphate regulation and/or bone mineralisation.

*Phex*-specific models of XLH include Ska1 mice, which contain a chemically-induced point mutation in a splice donor site just after exon 8 [75], *Phex*K496X (Jrt) mice, which contain a stop codon at amino acid 496 [76], and Hyp-2J and Hyp-Duk mice [77], which contain larger frameshift deletions. Phenotypic differences between Hyp-2J and Hyp-Duk mice have been observed and are discussed below. The Hyp-Duk mutation can result in the production of truncated PHEX proteins, which may retain some functions of PHEX [62].

Despite the range of animal models available, major challenges remain for elucidating the pathogenesis of XLH including: the low disease prevalence, the complexity of FGF23-related molecular networks, the diversity of *PHEX* mutations, the potential for residual PHEX function, and the potential impact of random X-inactivation on the severity of the female phenotype. Considering these challenges, known and proposed role(s) of FGF23 in XLH sequelae are discussed below.

### The role of FGF23 in XLH pathogenesis

When FGF23 was first described as the causative agent of ADHR, the authors commented on the similarities between ADHR and other diseases of inherited hypophosphatemia, including XLH [51]. The relationship between FGF23 and the pathophysiology of diseases of inherited hypophosphatemia has since been well-studied by comparing phenotypes of patients with FGF23-high and FGF23-normal hypophosphatemias (Table 1) to animal models (Table 2), healthy controls, and cases where FGF23 levels have been lowered, including blocking and knock-out experiments.

### Multiple pathways link elevated FGF23 to long bone abnormalities

Abnormal, disproportionate growth is a definitive feature of XLH and is primarily seen in reduced growth of endochondral long bones. Within the first few months of life, signs and symptoms of the disease become evident. Uncontrolled rickets and osteomalacia contribute to continuously diminished leg growth, which leads to short stature with elevated sitting height index (i.e. ratio between sitting height and stature), gait abnormalities due to deformities and muscle weakness, bone pain, deformity of weight-bearing limbs, with the development of Looser zones becoming evident in the mature skeleton (Fig. 1) [9, 78].

Briefly, endochondral bones develop from cartilaginous precursors that mineralise outwards from ossification sites. The cartilage remaining between ossification sites continues to grow, forming epiphyseal growth plates composed of germinal, proliferative and (upper and lower) hypertrophic zones. After completing active mitosis in the proliferative zone, epiphyseal chondrocytes of the upper hypertrophic zone enlarge and form columns that lengthen the developing bone, while chondrocytes of the lower hypertrophic zone mineralise the surrounding matrix and produce vascular endothelial growth factor that attracts invading vessel and bone cells [79]. The terminal hypertrophic chondrocytes undergo apoptosis and are replaced by osteocytes and osteoid that is then overlaid with hydroxyapatite to form mineralised bone [80, 81].

In hypophosphatemia, apoptosis of hypertrophic chondrocytes is arrested and is, by an unknown mechanism, followed by decreased chondrocyte proliferation and loss of organisation of the proliferative columns [80]. In addition, hypomineralisation of the newly formed bone leads to accumulation of osteoid and weakened bones. When pressure is applied to affected bones they bend under pressure, while loading appears to affect the function of hypomineralised growth plates, which collectively cause leg length to be more affected than arm span in XLH patients [57].

On the other hand, osteomalacia is caused by a generalised disruption of mineral deposition of newly-formed osteoid. Unlike rickets, which is a disease of the growth plates and thus only affects children, osteomalacia can affect both children and adults with XLH [81].

**Fig. 5:**
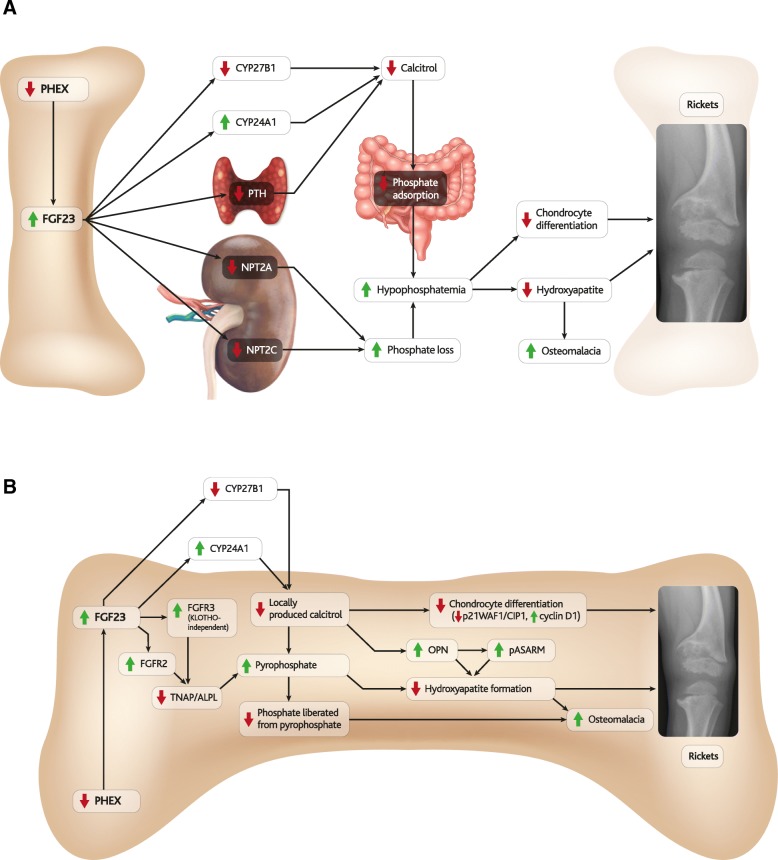
FGF23 and bone abnormalities. Schematic illustrations of (**a**) hypophosphatemic and (**b**) autocrine/paracrine molecular pathways that have been proposed to link FGF23 to bone abnormalities in XLH

A role for FGF23 in the pathogenesis of rickets and osteomalacia in TIO was identified shortly after its discovery [82], and was supported by the consistent appearance of bone abnormalities in FGF23-high diseases and animal models (Tables 1 and 2). Furthermore, the skeletal phenotypes of Hyp mice in which *Fgf23* was also ablated (Hyp*-Fgf23*^-/-^) were more similar to *Fgf23*^-/-^ mice than to Hyp mice [83]. The molecular pathways connecting FGF23 to abnormal bone development have since been further elucidated and include hypophosphatemia-mediated pathways and autocrine/paracrine pathways, which are respectively illustrated in parts A and B of Fig. 5 [84, 85].

### Hypophosphatemia-mediated mechanisms of FGF23-induced bone abnormalities

Hypophosphatemia is the primary mechanism by which elevated serum FGF23 affects bone development. Excess FGF23 results in hypophosphatemia, whether induced by direct injection [86] or increased stability [87, 88] of FGF23 (*Fgf23*-TG), or by downregulation of FGF23-suppressors including PHEX [5, 25, 52].

Hypophosphatemia leads to rickets by inhibiting mineralisation and apoptosis of hypertrophic chondrocytes [89], yet the contributions of FGF23 to hypophosphatemia are mediated by a complex network of pathways that ultimately increase urinary wasting, due to downregulation of the renal sodium-phosphate co-transporters NPT2A and NPT2C [90, 91], and decrease intestinal absorption of phosphate (Fig. 5a) [80, 81].

NPT2A and NPT2C play different roles in mice and humans. While humans develop severe hypophosphatemia (HHRH) when *NPT2C* is lost, depletion of *Npt2c*-alone in mice has no apparent effect on phosphate metabolism [54]. Depletion of *Npt2a*-alone results in upregulation of *Npt2c* and a mild hypophosphatemia and bone phenotype [54]. Yet mice missing both *Npt2a* and *Npt2c* display severe hypophosphatemia and rickets, as well as hypercalciuria, indicating a functional redundancy that is not seen in humans [92]. Such inter-species variation in gene function is a challenge for translating the study of these genes [93].

Nevertheless, the relationship between FGF23 and these phosphate channels is clear; direct administration of recombinant FGF23 has been seen to reduce renal expression of NPT2A in mice [94], and renal expression of NPT2A and/or NPT2C is downregulated in FGF23-high mice (Hyp or *Fgf23*-TG) and in patients with XLH [3, 90, 95].

Increased FGF23 affects calcitriol synthesis and degradation, thus hindering its ability to counterbalance hypophosphatemia. FGF23 downregulates renal 1α-hydroxylase (CYP27B1) and stimulates expression of 24-hydroxylase (CYP24A1), which limits production and increases degradation of calcitriol [15]. FGF23 also suppresses the secretion of PTH, which would otherwise promote expression of calcitriol [96], reducing intestinal adsorption of phosphate [97]. PTH is suppressed *via* MAPK/ERK signalling both in KLOTHO-dependent and -independent manners involving activation of the calcineurin-nuclear factor of activated T-cells (NFAT) pathway [96].

Disturbed regulation of the physiological responses of calcitriol to hypophosphatemia, in addition to the downregulation of the renal phosphate transporter channels, thus contribute to hypophosphatemia in diseases of excess FGF23 such as XLH (Fig. 5a). FGF23-mediated upregulation of prostaglandin E_2_ (PGE2) *via* inhibition of proximal tubule phosphate transport may also contribute to hypophosphatemia [98, 99], but the supporting evidence is less clear, since these animal studies were not supported by a subsequent under-powered crossover study in children [100].

### FGF23-mediated autocrine/paracrine pathways linked to bone abnormalities

The discovery that achondroplasia (a well-known skeletal dysplasia in which serum phosphate is unaffected) is caused by activating mutations of an FGF23 receptor (FGFR3), indicated that FGF23-related pathways may affect skeletal development in phosphate-independent manners [101]. This prospect was supported when abnormal mineralisation of Hyp osteocytes was observed in a phosphate-normal *in vitro* environment [102].

It has since become evident that hypophosphatemia-independent autocrine/paracrine effects of FGF23 can be mediated by calcitriol and tissue non-specific alkaline phosphatase (TNAP) (Fig. 5b).

### Calcitriol-dependent pathways

A recent study linked autocrine/paracrine roles of locally-produced calcitriol to the FGF23-mediated regulation of chondrocyte differentiation and bone mineral deposition [15].

Despite displaying hypophosphatemia and low serum calcitriol, mice with elevated FGF23 (Hyp or *Fgf23*-TG) did not develop skeletal abnormalities when CYP24A1 levels were repressed, either in *Cyp24a1*-null mutants or following blocking with CTA102 [15]. It was hypothesised that mineralisation in the control animals was disrupted by FGF23-mediated activation of CYP24A1 degrading locally-produced calcitriol, and CYP24A1-antagonists were proposed as novel therapeutic agents for XLH [15]. Furthermore, regulation of local CYP27B1 has recently been shown to differ between bone and kidney and local regulation of calcitriol is generally poorly understood and may be affected in XLH [103].

Conversely, there is also evidence suggesting that vitamin D does not play a direct role in bone development. For example, an early study in which vitamin D-deficient rats were either given vitamin D and infused with saline, or infused with concentrations of calcium and phosphorus to maintain plasma concentrations equal to those in the vitamin D treated animals, indicated that vitamin D did not play a role in the density or calcium-phosphate ratio of bone [104].

Inhibition of bone-derived calcitriol may contribute to rickets by inhibiting chondrocyte differentiation through downregulation of p21Waf1/Cip1 pathways and upregulation of cyclin D1 [15]. Calcitriol has also been shown to directly affect expression of OPN, a known inhibitor of hydroxyapatite crystal formation, however studies have indicated that calcitriol can either induce upregulation [105] or downregulation [106] of OPN, and whilst much of the literature suggests that calcitriol induces upregulation of OPN, no conclusive studies are currently available.

The SIBLING protein OPN contains an ASARM peptide motif [106, 107], cleavage of which releases phosphorylated ASARM (pASARM) peptides which are also potent inhibitors of mineralisation. Loose pASARM peptides are directly and exclusively cleaved by PHEX [4, 9, 40, 108]. Although reduced PHEX-mediated cleavage of pASARM also acts as an FGF23-independent mechanism for contributing to bone abnormalities in XLH [109–112], this process is exacerbated by FGF23-induced upregulation of OPN [108].

Furthermore, the recent observation of impaired urinary OPN excretion in *Npt2a*^-/-^ mice could indicate another FGF23-mediated contribution to the pASARM-mediated demineralisation of bone [113]. However, evidence that ablating *Fgf23* can also lead to upregulation of OPN indicates that the relationship between FGF23 and OPN is poorly understood and is likely to be complex [114].

### TNAP-dependent pathways

Recent evidence suggests that accumulation of pyrophosphate (PPi) may also play a role in impaired mineralisation in XLH.

Downregulation of TNAP (expressed by *Alpl*) suppresses hydrolysis of PPi and has been linked to post-natal mineralisation defects and hypophosphatasia, a normophosphataemic disease with rickets like bone abnormalities resembling those observed in XLH [115–117]. PPi is also a known mineralisation inhibitor, binding to and inhibiting formation of hydroxyapatite crystals [106, 107, 118].

A study comparing mice in which *Fgf23*, *Klotho* and/or vitamin D receptor (*Vdr*) genes were knocked out connected this TNAP-dependent pathway to FGF23, as accumulation of PPi was promoted in the osteoblastic cells of *FGF23*^*-/-*^ mice [49] .

The proposed autocrine/paracrine role of FGF23 was observed to act through TNAP and PPi in Hyp mice, whose osteoblasts and osteocyte-like cells were cultured *in vitro* and *ex vivo* and were compared to sections of Hyp bone [7]. Despite being separated from the *in vivo* hypophosphatemic environment, TNAP was inhibited and mineralisation defects arose in osteocyte-like cells, where PPi deposition was promoted and hydroxyapatite-formation was blocked [7].

That low levels of TNAP expression were observed in osteocyte-like cells but not in osteoblasts was further investigated and TNAP expression was found to correlate inversely with FGFR3 expression levels [7]. Chondrocyte proliferation has also been suppressed by FGFR3 activation *in vivo* and *in vitro*, resulting in suppression of linear bone growth [119]. Activation of FGFR3 can also lead to achondroplasia, which results in a more extreme disproportionate short stature than XLH. Experiments blocking FGF23 or FGFR3 demonstrated that FGF23 was suppressing TNAP transcription *via* KLOTHO-independent, FGFR3 signalling and ultimately leading to mineralisation defects [7]. This KLOTHO-independent pathway may explain findings that the overexpression of FGF23 can suppress osteogenesis in osteoblastic cells, which do not express KLOTHO [120].

Another key FGF23 receptor, FGFR2, also plays an important role in skeletal development [121], and was upregulated in the bones of Hyp mice [122]. There is a line of evidence connecting over-expression of FGFR2 in long bones to weakened long bones [123] *via* suppression of TNAP [123] and the production and accumulation of PPi [124, 125] (Fig. 5b).

Interestingly, the autocrine/paracrine effects of FGF23 appear to vary between mouse models of XLH. Although Jrt mice display growth retardation, skeletal abnormalities, hypophosphatemia and increased serum FGF23 and ALP levels similar to other murine models of XLH; unlike those models Jrt osteoblasts *in vitro* have been observed to resemble those from wild type males with respect to cellular differentiation and calcium deposition into bone matrix [76].

The osteoblast abnormality in Jrt mice may arise from a reduction in phosphate sensitivity mediated by *Phex* independently of FGF23 [126]. While hemizygous *Phex-*/Y mice (100% of cells carry one mutant *Phex*) displayed comparable skeletal abnormalities to heterozygous *Phex*-/*Phex*+ mice (50% of cells carry one mutant *Phex*), homozygous female *Phex*-/*Phex-* mice (100% of cells carry two mutant *Phex*) displayed exaggerated abnormalities, despite having equivalent serum phosphate and FGF23 concentrations [127]. Skeletal abnormalities in these animals therefore appeared to correlate with the dosage of *Phex* mutations, which may affect random X-chromosome inactivation or the sensitivity of osteocytes to serum phosphate and/or FGF23.

Jrt mice are an interesting model of XLH, and should be included in future studies investigating the roles of PHEX and FGF23 in the disease.

### FGF23-blocking ameliorates bone abnormalities

Finally, the relevance of FGF23 to bone abnormalities in XLH and other diseases of hypophosphatemia has been indicated by various animal experiments and clinical trials. For example, complete resection of FGF23-producing tumours has resolved hypophosphatemia, osteomalacia, bone pain and improved other skeletal manifestations in TIO [128, 129] and administration of FGF23-blocking antibodies has improved growth retardation of juvenile Hyp mice, accelerating weight gain, increasing tail length, decreasing osteoid volume and thus improving bone mineralisation while improving elongation of femoral and tibial bones [130–132]. FGF23 antibodies have also improved levels of serum phosphate, serum calcitriol, and alkaline phosphatase, as well as rickets severity (RSS), radiographic global impression of change (RGI-C), and Western Ontario and the McMaster Universities Osteoarthritis Index (WOMAC) scores in clinical trials involving paediatric or adult patients with XLH (Paediatric trial: NCT02163577; Adult trial NCT02526160) [133–135].

### Ectopic calcification and ossification in XLH

**Fig. 6:**
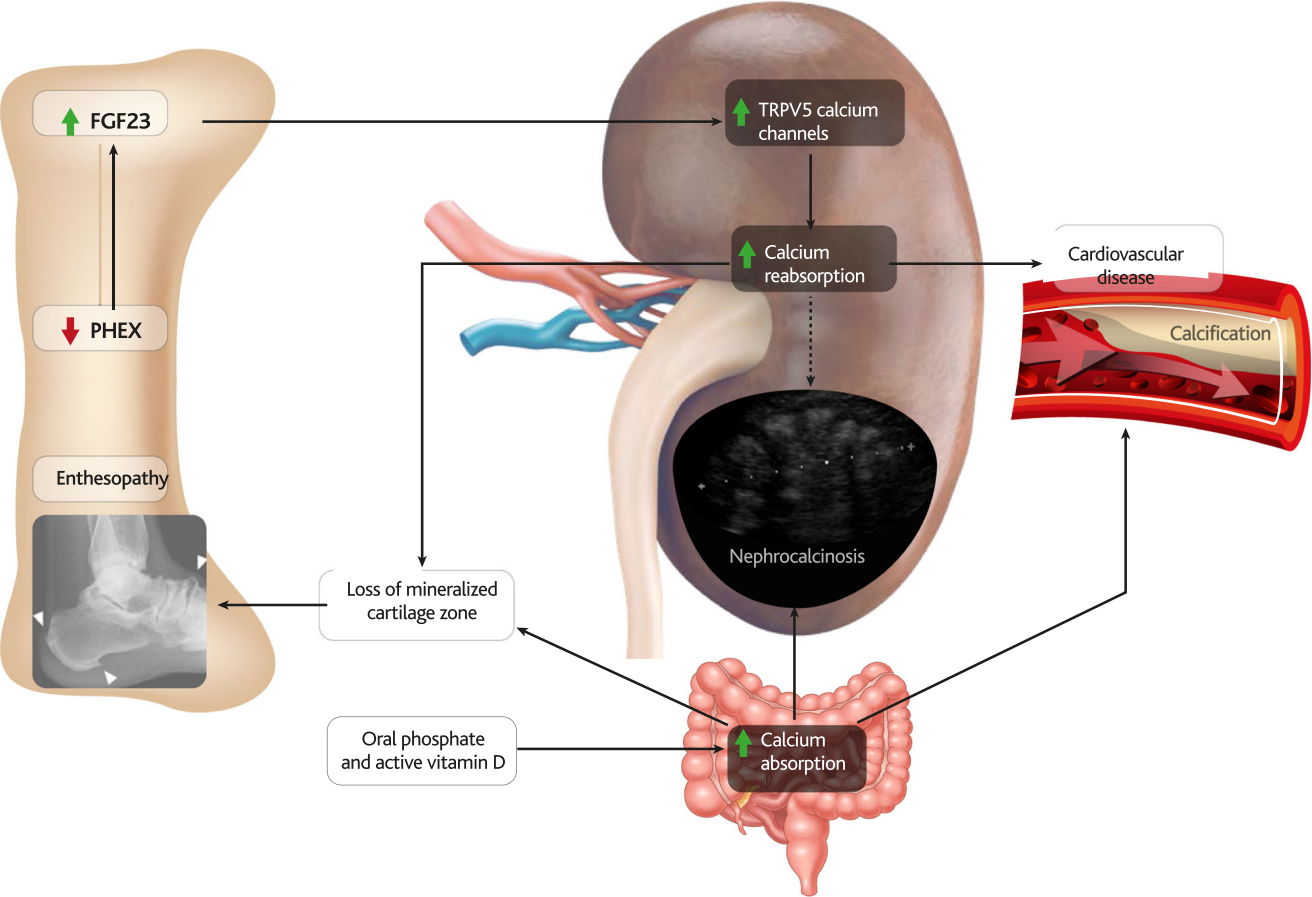
FGF23 and kidney abnormalities. Elevated levels of FGF23 in XLH increase renal expression of TRPV5 calcium channels, contributing to local excesses of calcium and general hypercalcemia, which may contribute to enthesopathies and calcification of arteries, resulting in hypertension and cardiovascular disease. Although nephrocalcinosis occurs in XLH as a side-effect of conventional therapy, the TRPV5-mediated increase in renal calcium absorption may play a permissive role

Ectopic calcification in XLH can affect the kidneys (nephrocalcinosis), joints, and bone attachments of tendons (enthesopathies) (Fig. 6). While nephrocalcinosis has long been considered a side effect of conventional treatment [136], enthesopathies have also been observed in untreated patients [137].

### Nephrocalcinosis

Nephrocalcinosis has been observed in as few as 22% and as many as 100% of XLH patients [66, 138, 139]. This variation can be partially attributed to small study sizes and high patient and treatment variability.

Nephrocalcinosis is often composed of calcium phosphate precipitation and is thought to be due to one or a combination of hypercalciuria, hyperphosphaturia, hyperoxaluria, and secondary hyperparathyroidism [140–142]. Nephrocalcinosis has not been reported in untreated XLH patients and is widely considered to be a result of conventional therapy [143] associated with active vitamin D dosage [144]. In addition, other soft-tissue calcifications such as ocular, myocardial, and aortic valve calcification have been reported in XLH patients with persistent secondary or tertiary hyperparathyroidism and/or high dose calcitriol and phosphate treatment [145].

The hypercalciuric properties of NPT2A/C downregulation, alongside the effects of FGF23 on NPT2A/C expression, have been briefly discussed above, and the impact of NPT2A/C impairments in patients are further explored in the paper by Bergwitz and Jüppner [146]. Dysfunctions in NPT2A alone have been associated with severe renal calcification [147], whilst mutations in *NPT2A* and *NPT2C* have also been reported in patients with renal stone disease and nephrocalcinosis [148, 149].

A recent study has also shown that upregulation of OPN *via* FGF23/PHEX can contribute to nephrocalcinosis and nephrolithiasis observed in mice on a high-phosphate diet [113].

A potential role for FGF23 in enhancing renal calcium reabsorption has also been observed in XLH [150], which may be mediated through the transient receptor potential cation channel subfamily V member 5 (TRPV5) channel, which promotes cellular uptake of calcium and therefore calcification [151, 152].

Excessive mineralisation also occurs in the heart and kidney of mice in which *Fgf23* has been ablated, regardless of whether or not the mice possessed the Hyp mutation [83]. This further illustrates the suppressive effect FGF23 has on mineralisation.

Nephrocalcinosis has not been observed during FGF23-blocking trials, probably because the treatment does not include active vitamin D [130]; however, long-term data are lacking.

### Cardiovascular calcification and hypertension

Reports of cardiovascular abnormalities and hypertension in patients with XLH are rare, inconsistent, and considered to be side effects of conventional therapy and/or FGF23-driven increased renal sodium reabsorption [14, 66, 139, 152, 153]. Studies have reported hypertension [66] and left ventricular hypertrophy in only a minority of subjects [139], or found no evidence of cardiovascular myocardial dysfunction symptoms in any of 11 XLH patients [154].

A recent study of XLH patients found hypertension correlating with reduced estimated glomerular filtration rate (eGFR) in 6/22 patients, most of whom also had secondary hyperparathyroidism [66]. The authors were unable to determine whether hypertension was a primary consequence of XLH or a secondary consequence of conventional therapy and concluded that “multiple factors” presumably played a role [66].

The over-expression of FGF23 has also been associated with various aspects of cardiovascular disease in chronic kidney disease (CKD) including cardiomyocyte hypertrophy, vascular calcification, stroke, and endothelial dysfunction [155–160]. Atherosclerosis has been proposed as a mechanism by which FGF23 may promote cardiovascular events and stroke in these patients [157]. However, the pathogenic mechanism is unlikely to be mediated by KLOTHO, which is excreted by the CKD-affected kidney [161]. FGF23 may instead contribute to cardiovascular disease in CKD by directly interacting with FGFR4 on cardiomyocytes to induce cardiomyocyte hypertrophy [162, 163] or with hepatocyte FGFRs to induce hypertension [164] which can lead to blood vessel calcification [165, 166].

In addition, FGF23 was shown to directly regulate the membrane abundance of the Na(+):Cl(-) co-transporter NCC in distal renal tubules by a signalling mechanism involving the FGF receptor/αKlotho complex [152]. This suggests that FGF23 is a key regulator of renal sodium reabsorption and plasma volume and may explain the association of FGF23 with cardiovascular risk in CKD patients. In addition, FGF23 is also generally associated with the progression of CKD [167]. Nevertheless, FGF23 levels in CKD are elevated well above those observed in hereditary hypophosphatemia and at those concentrations FGF23 may reach toxic levels that are not relevant to XLH [168].

### Enthesopathy

In patients with hereditary hypophosphatemia, inappropriate mineralisation of fibrocartilage can develop where tendons insert into bone (entheses). The developing spurs (enthesophytes) can then cause joint stiffness and pain (enthesopathy), which often affects patients with XLH [138]. Mineralising enthesopathies of fibrocartilaginous insertion sites affect a majority of ankles in patients with XLH, and appear to be strongly correlated with increasing age [2, 169, 170]. Enthesopathies are also commonly observed in other phosphate-wasting disorders of excessive FGF23 (ARHR1, AHRH2), and in murine models of XLH (Hyp, *Fgf23*-TG) [171–173].

The initiation of mineralising enthesophytes at the bony insertion site is poorly understood, but is thought to occur following degeneration of mineralised cartilage during insertion site development [174]. Expression of *Fgfr3* and *Klotho* in murine fibrocartilage cells indicates that they are likely to be directly affected by FGF23 [169].

Fibrocartilage is composed of an uncalcified zone containing alkaline phosphatase-negative chondrocytes, and a calcified zone where chondrocytes express alkaline phosphatase and are surrounded by a mineralised matrix that covers the bone surface. Significantly greater numbers of alkaline phosphatase-positive fibrocartilage cells have been observed in joints of Hyp mice than in control mice, yet the typically narrow mineralised zone was also completely lost [169]. The observed fibrochondrocyte hyperplasia was proposed to pre-date loss of the mineralised zone and to cause enthesopathy [169].

Enthesopathies have been observed in untreated XLH patients [137, 175] and they have been reported to be unaffected by conventional therapy in XLH patients [176]. In Hyp mice conventional therapy has not only failed to ameliorate fibrochondrocyte hyperplasia, but also exacerbated mineralisation of enthesopathies [171].

Development of enthesopathies may be mediated by matrix metalloproteinase 13 (MMP13), a gene that prepares cartilage matrix for calcification [177] and a critical target gene during the progression of osteoarthritis.

Expression of MMP13, FGF23 and OPN are all downregulated in enthesopathic Hyp cartilage, but not in the osteoblasts of those same mice. This indicates that the downregulation of these genes is chondrocyte-specific and may indicate that the hyperplastic chondrocytes observed in enthesopathies are immature [174]. This observation also highlights the importance of assessing gene expression levels of specific cell types where possible, rather than relying on serum levels to develop mechanistic models.

Enthesopathic sites in Hyp and *Fgf23*-TG mice also displayed increases in sulphated proteoglycans [171]. The cushioning effect of the sulphated proteoglycans combined with the greater surface area of XLH bones are thought to stabilise and protect joints from the abnormally high compressive forces exerted through weakened and misshapen long bones [171]. Mineralising enthesopathies may therefore be a secondary effect of long bone hypomineralisation causing weaker and more bendable bones, with potentially more strain on the entheses and their attachment. Therefore, correcting gross skeletal abnormalities and restoring normal biomechanics may theoretically contribute to correction of enthesopathy.

Although the effects of FGF23 blocking on enthesopathies have not specifically been assessed, significant improvements have been observed in treated XLH patients scored on the Western Ontario and McMaster Universities Osteoarthritis Index (WOMAC), which focuses on patient perception of joint pain [178].

### Skeletal muscle defects in XLH

Muscle pain or weakness has been reported by a majority of adult hereditary hypophosphatemic rickets patients in one study [138], and Hyp mice display reduced grip strength and spontaneous movement compared to controls [131].

Despite having normal muscle size, and in the absence of leg deformities, subjects with hereditary hypophosphatemic rickets had lower muscle density and lower peak muscle force and power compared to age- and gender-matched controls [179, 180]. Since muscle force is strongly correlated with bone strength, and osteocytes have been connected to muscle mass and function *via* mechanical loading, PGE2 and Wnt3a [181], the abnormal skeletal phenotype in XLH patients may contribute to skeletal muscle defects.

It is also interesting to note that some studies have found strong correlations between extremes in phosphate levels and impaired muscle force, whilst others have found that hypophosphatemia appears to be associated with muscle weakness. These findings suggest that the development of the skeletal muscle defects observed in patients with XLH may be multifaceted [182, 183].

Skeletal muscle wasting, weakness and pain have also been observed in patients with TIO [128, 129, 184]. The lack of either skeletal abnormality or genetic mutation in these patients indicates that FGF23 may contribute to the development of these manifestations either directly or *via* hypophosphatemia.

The phosphaturic actions of FGF23 may contribute to the muscular phenotype by decreasing muscle ATP synthesis and causing muscle weakness, which has been observed in both HHRH patients and *Npt2a*^-/-^ mice [185]. This correlation is supported by evidence that phosphate supplementation has reversed skeletal muscle abnormalities in a case of chronic fatigue [186] and reversed muscle weakness in a patient suffering FGF23-induced hypophosphatemic osteomalacia [187]. Phosphate supplementation has also ameliorated post-operative weakness and muscle tremors in a dog [188] and vitamin D-deficiency-induced muscle weakness in rats [182].

Collectively, these results indicate that FGF23-induced hypophosphatemia is associated with muscle weakness in XLH. However, the expression of PHEX in myocytes indicates the potential for a more direct role for FGF23 in muscular weakness in XLH [74], and FGF23 has been shown to induce senescence in mesenchymal stem cells derived from skeletal muscle [189].

On the other hand, there may be a role for exercise-stimulated FGF23 in controlling production of ROS production and enhancing mitochondrial function [190]. Although FGF23 levels are likely to be consistently higher in XLH than during exercise, the study by Li et al identifies a novel effect of FGF23 on skeletal muscle, which may be induced by the high FGF23 levels in XLH. Plasma levels of FGF23 are also positively associated with muscle mass in haemodialysis patients, which could indicate a role for FGF23 in improving muscle strength [191].

Nevertheless, resection of an FGF23-producing tumour has resolved muscle pain in a patient with TIO [128] and therapeutic application of an FGF23-blocking antibody has increased grip strength and spontaneous movement in Hyp mice [131] and led to full recovery of bone and muscle pain when treating patients with iron-induced FGF23-mediated hypophosphatemic osteomalacia [192].

### Craniosynostosis in XLH

**Fig. 7:**
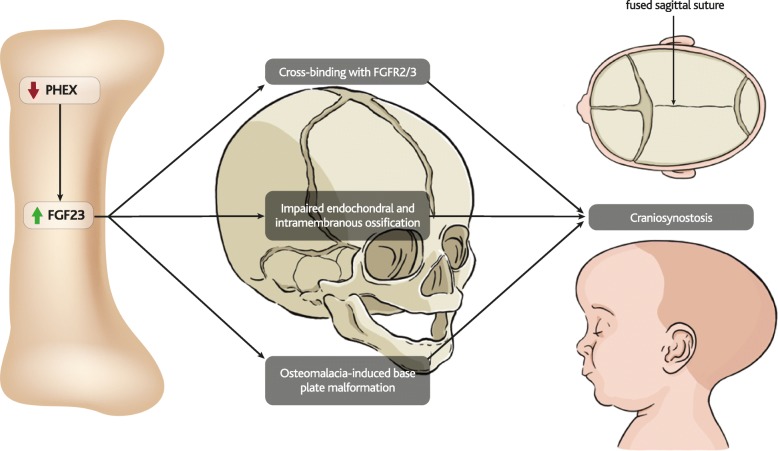
FGF23 and cranial abnormalities. Over-expression of FGF23 leads to up-regulation of FGFR2/3 signalling. Cross-binding of FGF23 with FGFR2/3 at cranial sutures, impaired endochondral ossification of the skull, and osteomalacia-induced malformation of the base plate can all lead to craniosynostosis

Craniosynostosis is a cranial malformation that results from premature fusing of the cranial sutures during development (illustrated in Fig. 7). Beyond abnormal skull shape, craniosynostosis and defective mineralisation in XLH patients can be associated with Arnold-Chiari malformations, which may cause central nervous system problems [193] . Hypophosphatemic rickets has long been linked to craniosynostosis [194], but the relationship is poorly understood [195].

Cranial malformations arise in a range of diseases that involve activation of FGF23 receptors, including osteoglophonic dysplasia (OGD) (FGFR1, [196]), Crouzon and Apert syndromes (FGFR2, [44]) and achondroplasia (FGFR3, [197]). Achondroplasia affects the size and shape of the cranial base, as well as reducing length of the nasal bone [198], which has also been observed in patients with hereditary hypophosphatemia [199] and Hyp mice [200].

Overexpression of FGFR2 and FGFR3 have been shown to affect both intramembranous and endochondral ossification in the skull [123, 197]. Beyond downregulation of TNAP, proposed mechanisms for these changes include TGF-β/BMP signalling (ERK1/2) and Wnt signalling [125, 201, 202], while cross-binding of FGF23 with FGFR2 and FGFR3 at the cranial sutures has also been proposed to contribute to craniosynostosis [203]. However, it remains difficult to isolate these pathways as specific mechanistic links to craniosynostosis because they have also been linked to bone mineralisation.

Effects of blocking FGF23 on the development of craniosynostosis have not been reported at this stage [130, 131, 204].

### Dental defects in XLH

Despite an outwardly normal dental appearance, severe dental disease including dental abscesses, periodontal problems and malocclusion [205] has been observed in as many as 75% of untreated XLH patients [206].

**Fig. 8:**
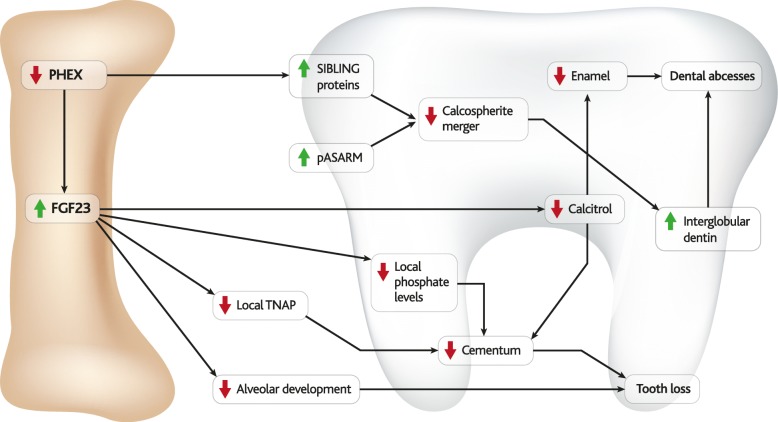
FGF23 and dental abnormalities. A schematic representation of molecular pathways that have been proposed to contribute to dental manifestations of XLH

Teeth are primarily composed of three layers, the internal pulp is surrounded by dentin, which is itself coated on the crown by enamel and on the root by cementum. Although the dental manifestations of XLH have been well-reviewed, the responsible molecular mechanisms are poorly understood [205]. Proposed mechanisms are illustrated in Fig. 8.

### Normal development of dental tissues

During normal dental development, collagenous predentin is deposited by odontoblasts and matures into dentin through two phases. The first phase involves formation of calcium hydroxyapatite crystals as globules (or calcospherules) in the predentin collagen fibres. The second phase involves deposition of new areas of mineralisation that are layered onto the original crystals, expanding them almost to the point of fusion.

Enamel formation (amelogenesis) commences after the first layer of dentin has been deposited and continues in repeated stages of secretion and maturation. The development of enamel and dentin is thereafter mutually induced, while deposition of cementum by cementoblasts occurs later in tooth development.

### Dentin defects

Although some incompletely-crystallised interglobular dentin usually remains in the spaces between fully-formed hydroxyapatite crystals, excess interglobular dentin is a marker of certain dental abnormalities.

Severe under-mineralisation of circumpulpal dentin is a hallmark of untreated children with XLH, whose teeth contain large interglobular spaces, enlarged pulp chambers, and prominent pulp horns that extend to the dentino-enamel junction [207, 208]. The porous nature of dentin in children with XLH makes their teeth prone to bacterial invasion, abscesses, and necrosis [208], which often occur “spontaneously” in the absence of previous damage [112].

The presence of FGF23 mRNA in ameloblasts and odontoblasts, along with observations of significant reductions in mineral density, dental volume, and reparative dentin area in *Fgf23*-TG mice indicates that FGF23 may be directly involved in dentinogenesis and mineralisation [209], as do observations of excess interglobular dentin in Hyp and *Fgf23*-TG model mice from an early age [209, 210].

Contrasting the high frequency of dental abscesses in Hyp mice [211] with their relative absence in *Phex*- and *Fgf23*-normal hypophosphatemic mouse models [212] or in HHRH patients [213, 214] indicates that a phosphate-independent mechanism is likely to contribute to formation of dental abscesses in XLH.

Furthermore, spontaneous dental abscesses have not been reported as clinical features of TIO and iron-induced osteomalacia, which are FGF23-high diseases of hypophosphatemia that develop later in life. This may indicate that hereditary hypophosphatemia and/or other effects of FGF23 affects formation of dentin and enamel structures during early dental development. Accordingly, treatment with calcitriol and phosphate supplementation during the early time window of dental development correlates with improved dental health later in life [208, 215, 216].

### Enamel defects

Enamel defects observed in XLH patients include microclefts and irregular surface structure, through which bacteria could invade the tooth and form abscesses [217].

Evidence from rodent models indicates that enamel phenotypes in XLH may be phosphate-independent and mediated by calcitriol *via* osteocalcin [218–220]. However, reports of dental osteocalcin levels vary between Hyp and *Fgf23*-TG mouse models of XLH, which have been reported to respectively up- and down-regulate osteocalcin compared to wild type mice [209]. If these results are accurate, there may be an FGF23- and calcitriol-independent role of PHEX in mediating osteocalcin deposition.

### Cementum defects

Loss of dental attachment is common in XLH, and can result from defects in cementum, periodontal ligament and/or alveolar bone [216].

Studies of Hyp and *FGF23*-/- mice have identified a role for FGF23 in the development and maintenance of the dentoalveolar complex [112, 221], and cementum has been observed to be thinner in Hyp mice than in wild type controls, with discontinuous mineralisation and a globular appearance [210].

A variety of molecular mechanisms have been proposed to contribute to cementum defects in XLH, including sensitivity to local levels of phosphate [222] and to altered regulation of TNAP [223]. Bone-targeted TNAP has also rescued defects in cementum and alveolar bone in patients with hypophosphatasia [205].

Calcitriol has also been reported to affect murine cementogenesis in a DMP1- and FGF23-mediated manner [224]. A role for FGF23 in regulation of cementum is also indicated by the levels of bone sialoprotein (BSP) (decreased) and DMP1 (elevated) in the cementum of Fgf23^−/−^ mice [225]. Furthermore, in human studies it has been shown that early childhood initiation and long-term persistence of conventional XLH therapy into adulthood improves the periodontal deformities typical of XLH, likely as a result of cementum and dentin defect correction [216].

Despite the differences between tooth and bone development, mineralisation of both tissues involves similar molecular processes and is often affected by similar molecular mechanisms [205]. However, the effects of blocking FGF23 on dental development – of particular interest for treatment of patients with XLH – have not been reported at this stage [130, 131, 204].

### Hearing loss

Patients with XLH have been observed to experience hearing loss affecting low and high frequencies, which can be associated with tinnitus and vertigo, and has been compared to symptoms of endolymphatic hydrops (ELH) [226–228].

**Fig. 9:**
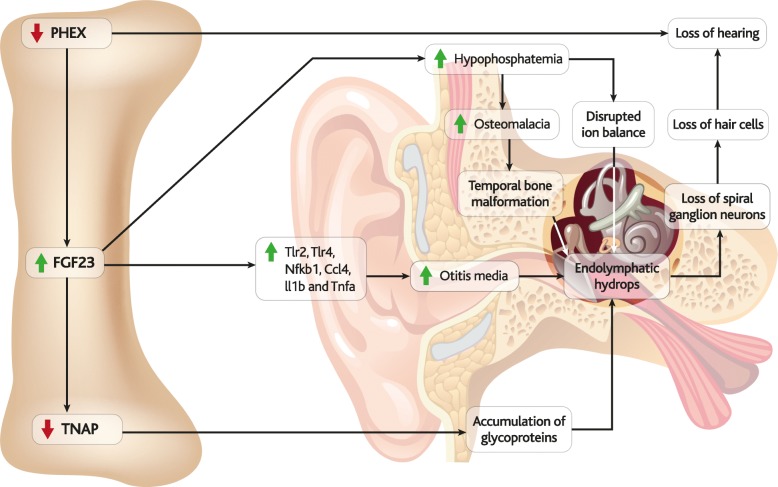
Mechanisms of hearing loss in XLH. A schematic illustration of the structure of the ear is overlaid with the molecular pathways that may connect FGF23 to hearing loss in XLH

Molecular mechanisms that have been reported to contribute to hearing loss in XLH are depicted in Fig. 9, yet the aetiology of endocrinological and metabolic hearing loss is complex [229, 230], and the literature can be even more difficult to interpret than for other manifestations. As such, more work is needed to fully elucidate the molecular links between FGF23, XLH and hearing loss.

### Hearing Loss and XLH

Reports of hearing loss in XLH patients are variable depending on the age and selection criteria of the cohort and range from 16% of subjects with hypophosphatemic bone disease experiencing sensorineural hearing loss [231], to 76% of subjects with X-linked hypophosphatemic osteomalacia experiencing hearing loss as detected by pure tone audiometry [226]. In the latter study, subjective hearing loss was reported by 48% of subjects.

Other studies have assessed conductive hearing loss [226], sensorineural hearing loss [231], cochlear dysfunction [232] and electrical activity in the auditory centres of the brain [230] with widely varying results. A more standardised approach for assessing hearing loss is clearly required for future studies.

A low prevalence of hearing loss has been reported in many studies of XLH patients, and the cause of hearing loss is often confounded by age, other genetic or environmental factors. Hearing loss in XLH patients and in general has been observed to occur in certain families [232], or in older patients who had experienced excessive noise exposure [231]. The prevalence of hearing loss has also been observed to vary between mouse models of XLH with different *Phex* mutations, genetic backgrounds or sex [77, 233]. These confounding factors make it difficult to identify mechanisms that contribute to hearing loss in XLH.

Hearing loss was first associated with specific *Phex* mutations when Hyp mice (in which ear-related phenotypes had not been observed) were compared to Gy mice (who were commonly deaf) [70]. Potential contributions of PHEX and FGF23 to hearing loss were confounded because both mutations extended beyond the *Phex* coding region (Table 2), and Gy mutations affected the nearby *SmS* gene, which has been associated with hearing loss [234]. The role of FGF23 in XLH-related hearing loss should instead be studied using animal models with *Phex*-specific mutations like Hyp-Duk, Hyp-2J, and Ska1 mice.

Of the *Phex*-specific models, male Hyp-Duk mice commonly displayed hearing loss, but Hyp-2J mice did not [77]. Furthermore, the prevalence of hearing loss in Hyp-Duk mutants decreased when the mice were cross-bred onto different strains [233]. These confounding effects of specific mutation and genetic background on XLH-related hearing loss would be further compounded when studying genetically diverse patients with XLH that have various *PHEX* mutations. Nevertheless, these findings have led the BALB/cUrd strain of Hyp-Duk mice being used as a model for studying the natural history of ELH [230, 233, 235].

### Endolymphatic hydrops

ELH has been associated with damage to the organ of Corti and spiral ganglion neurons (SGNs) and is the most well-studied mechanism of hearing loss in XLH [226, 236]. The severity of ELH has also correlated with the severity of hearing loss in Hyp-Duk mice [237].

ELH is caused by an inappropriate volume or composition of endolymph within the inner ear, and hearing loss followed by neuronal loss and then hair cell loss is commonly observed in models of ELH [238, 239]. In the Hyp-Duk model, ELH developed by P21 (21 days after birth), SGN in the organ of Corti were lost by around P90, and morphologically abnormal hair cells arose much later (>P300) [239]. SGN loss in ELH progresses from the apical to the basal cochlear turn and is at least partially caused by apoptosis [77, 233, 239]. It is unclear whether the relationship between ELH and SGN is causative or correlative, although it has been suggested that cell stress caused by elevated pressure associated with hydrops may contribute to the observed apoptosis [239]. The cause of ELH in XLH is also unclear [240].

With regards to the volume of endolymph, hearing loss in the Hyp-Duk model of XLH does not consistently correlate with increased endolymph space [77, 233], with morphological abnormalities of the surrounding temporal bone [77, 233], or with obstruction of the endolymphatic duct [233]. Disruption of periductal channels embedded in the temporal bone is therefore a candidate for contributing to ELH [230, 241], but has not yet been studied in XLH.

Furthermore, although conventional therapy has been observed to improve the bony structure surrounding the ear and prevent osteoid deposition, the treatment did not prevent ELH or hearing loss [230]. Unfortunately, in this study mice were under-dosed with phosphate and other symptoms were also unaffected, so a follow up study would be required to test these findings [230].

The chemical composition of endolymph in XLH could be altered by varied aural expression of ion channels [240] or through metabolic interactions with the surrounding phosphate-deprived bone [230]. A disrupted chemical composition could also be linked to aural precipitates that have been observed in various animal models of XLH [77, 242]. Characterising the endolymphatic fluid and any precipitate in XLH patients would shed light on this matter.

### Inflammation and hearing loss

The reported formation of perilymphatic precipitate and of inappropriate bone formation in the membranous labyrinth (a potential mechanistic parallel to nephrocalcinosis or enthesopathy in XLH) [243] may also contribute to inflammation (otitis media and serous labyrinthitis), which has in turn been linked to ELH [244] and to hearing loss in XLH [62, 233, 243].

However, these results have all come from animal studies, and otitis media has not been observed in XLH patients [62]. To determine if these animal models are suitable, XLH patients that experience hearing loss should therefore be assessed for inflammation and have their DNA sequenced for mutations that resemble Gy or Hyp-Duk.

### Other XLH manifestations

Patients with XLH rarely report symptoms related to a weakened immune system, however FGF23 has been connected to the innate immune system in CKD, impairing neutrophil recruitment [44, 245] and the antimicrobial molecule LL37 synthesis in peripheral blood mononuclear cell monocytes [246]. FGF23 has also been connected to increased deaths by infectious diseases [247]. As these sequelae have not been connected to XLH, they are beyond the scope of this article, and we refer to a recent review of the subject [246].

## Conclusions

Since FGF23 was identified as the causative agent of ADHR and TIO, it has been shown to play a key role in the pathology of XLH and most other inherited hypophosphatemic diseases. This review has described literature exploring the mechanisms by which excess FGF23 contributes to the clinical manifestations and morbidity of XLH.

There has been considerable advancement in the understanding of XLH pathogenesis over the last two decades. Indeed, most manifestations of XLH are now known to be caused by FGF23-induced hypophosphatemia resulting from downregulation of sodium-phosphate transporters in the renal distal tubule, and repression of serum calcitriol. In addition, local repression of calcitriol and TNAP may also inhibit mineralisation *via* FGF23-mediated upregulation and loss of PHEX-mediated degradation of OPN and pASARM, alongside accumulation of PPi.

These roles of FGF23 in XLH pathology have been further demonstrated by anti-FGF23 antibody treatment, which can normalise phosphate and vitamin D metabolism and improve rachitic changes in XLH patients [133] and Hyp mice [130–132].

To further elucidate the role of FGF23 in manifestations of XLH, it is important to clearly define and compare the causes and manifestations of other diseases of hypophosphatemia and their representative animal models. Studies assessing individual manifestations of XLH are often under-powered and generate vastly different estimates of frequency, intensity, and correlation with specific genotypes.

Larger patient registries and multicentre studies that include greater numbers of XLH patients are needed to further clarify the prevalence, phenotypic spectrum, genotype-phenotype correlation, and response to treatment of patients with XLH.

## Abbreviations

7B2: Neuroendocrine protein 7B2
ADHR: Autosomal dominant hypophosphatemic rickets
ALP: Alkaline phosphatase
ALPL: Alkaline phosphatase gene
ARHR: Autosomal recessive hypophosphatemic rickets
ASARM: Acidic serine aspartate-rich-MEPE-associated protein
ATP: Adenosine triphosphate
BALB/cUrd: BALB/cAnBomUrd-Foxn1nu
BSP: Bone sialoprotein
Calcitriol: 1,25(OH)_2_D, active vitamin D
CKD: Chronic kidney disease
Cyp24: Cytochrome P450 family 24 subfamily A member 1, CYP24A1
DMP1: Dentin matrix acidic phosphoprotein 1
ELH: Endolymphatic hydrops
ENPP1: Ectonucleotide pyrophosphatase/phosphodiesterase family member 1
Fam20C: Family with sequence similarity 20, member C
FGF: Fibroblast growth factor
FGF23Ab: Murine antibody against FGF23
FGFR: Fibroblast growth factor receptor
GACI: Generalised arterial calcification of infancy
GALNT3: Gene encoding Polypeptide N-acetylgalactosaminyltransferase 3
HHRH: Hereditary hypophosphatemic rickets with hypercalciuria
HRHPT: Hypophosphatemic rickets and hyperparathyroidism
HS: Heparan sulfate
kDa: Kilodalton
MEK-ERK: Mitogen-activated protein kinase kinase-extracellular signal-regulated kinases
MEPE: Matrix extracellular phosphoglycoprotein
MIM: Mendelian Inheritance in Man
MMP13: Matrix metalloproteinase 13
mRNA: Messenger ribonucleic acid
NADPH: Nicotinamide adenine dinucleotide phosphate
NFAT: Nuclear factor of activated T-cells
NFκB: Nuclear factor kappa-light-chain-enhancer of activated B cells
OGD: Osteoglophonic dysplasia
OMIM: Online Mendelian Inheritance in Man
OPN: Osteopontin
Orai1: Gene encoding calcium release-activated calcium channel protein 1
pASARM: Phosphorylated acidic serine aspartate-rich-MEPE-associated protein
PC2: Proprotein convertase, subtilisin/kexin-type 2
PC5/6: Proprotein convertase, subtilisin/kexin-type 5/6
PGE2: Prostaglandin E_2_
PHEX: Phosphate Regulating Endopeptidase Homolog, X-Linked
ppGalNAc-T3: Polypeptide N-acetylgalactosaminyltransferase 3
PPi: Pyrophosphate
PTH: Parathyroid hormone
RGI-C: Radiographic global impression of change
ROS: Reactive oxygen species
RSS: Rickets severity score
SGN: Spiral ganglion neurons
SIBLING: Small integrin-binding ligand, N-linked glycoprotein
SLC34A3: Solute carrier family 34 member 3
SmS: Spermine synthase
TGF-β/BMP: Transforming growth factor beta/bone morphogenetic protein
TIO: Tumor-induced osteomalacia
TNAP: Tissue non-specific alkaline phosphatase
TRPV5: Transient receptor potential cation channel subfamily V member 5
VDDR1A: Vitamin D dependent rickets type 1A
Vdr: Vitamin D receptor
WOMAC: The Western Ontario and McMaster Universities Osteoarthritis Index
XLH: X-linked hypophosphatemic rickets
αHIF-1: Hypoxia inducible factor-1 alpha
